# Mask Reuse in the COVID-19 Pandemic: Creating an Inexpensive and Scalable Ultraviolet System for Filtering Facepiece Respirator Decontamination

**DOI:** 10.9745/GHSP-D-20-00218

**Published:** 2020-09-30

**Authors:** Rachel M. Gilbert, Michael J. Donzanti, Daniel J. Minahan, Jasmine Shirazi, Christine L. Hatem, Brielle Hayward-Piatkovskyi, Allyson M. Dang, Katherine M. Nelson, Kimberly L. Bothi, Jason P. Gleghorn

**Affiliations:** a Department of Biomedical Engineering, University of Delaware, Newark, DE, USA.; b Department of Biological Sciences, University of Delaware, Newark, DE, USA.; c Department of Chemistry and Biochemistry, University of Delaware, Newark, DE, USA.; d Department of Chemical and Biomolecular Engineering, University of Delaware, Newark, DE, USA.; e Global Engineering, University of Delaware, Newark, DE, USA.

## Abstract

As the current COVID-19 pandemic illustrates, not all hospitals and other patient care facilities are equipped with enough personal protective equipment to meet the demand in a crisis. Health care workers around the world use filtering facepiece respirators to protect themselves and their patients, yet during this global pandemic they are forced to reuse what are intended to be single-use masks. This poses a significant risk to these health care workers along with the people they are trying to protect. Ultraviolet germicidal irradiation (UVGI) has been validated previously as a method to effectively decontaminate these masks between use. However, not all facilities have access to the expensive commercial ultraviolet type C (UV-C) lamp decontamination equipment required for UVGI. UV-C bulbs are sitting idle in biosafety cabinets at universities and research facilities around the world that have been shuttered to slow the spread of COVID-19. These bulbs may also be available in existing medical centers where infectious diseases are commonly treated. We developed a method to modify existing light fixtures or create custom light fixtures that are compatible with new or existing UV-C bulbs. This system is scalable; can be created for less than US$50, on site and at the point of need; and leverages resources that are currently untapped and sitting unused in public and private research facilities during the pandemic. The freely accessible design can be easily modified for use around the world. Health care facilities can obtain this potentially lifesaving UVGI resource with minimal funds by collaborating with research facilities to obtain the UV-C meters and UV-C bulbs if they are unavailable from other sources. Although mask reuse is not ideal, we must do what we can in emergency situations to protect our health care workers responding to the pandemic and the communities they serve.

## Abstract (precis)

We outline a simple, low-cost design—both scalable and adaptable worldwide—to decontaminate filtering facepiece respirators (FFRs) using ultraviolet bulbs and supplies found in most hardware stores. The setup will help health care workers safely reuse FFRs in light of the shortages during the COVID-19 pandemic.

## Key Messages

- Ultraviolet germicidal irradiation (UVGI) systems can be used to decontaminate filtering facepiece respirators that are in short supply during the current COVID-19 pandemic, but are costly and scarce.
- Custom-built UVGI systems can be easily and affordably created using common items found in hardware stores and within research institutions.
- Health care workers and administrators should consider this setup as a cost-effective option to combat personal protective equipment (PPE) shortages during the current pandemic.
- Academic institutions should consider fostering collaborations with local health care institutions to provide idle resources to front line health care workers facing PPE shortages.

## INTRODUCTION

Health care workers (HCWs) are critical to the care and treatment of individuals with the severe acute respiratory syndrome coronavirus 2 (SARS-CoV-2) or coronavirus disease known as COVID-19. In addition to the needed beds and ventilators, personal protective equipment (PPE), particularly filtering facepiece respirators (FFRs), are essential to ensure the health and safety of not only trained doctors, nurses, and emergency response personnel, but also other health care facility staff who play an important role in cleaning, disinfecting, and preparing spaces for patient care. Additionally, whereas much of the focus of HCW risk is on large hospitals and current hotspots that do not have access to FFRs, also called N95 masks in the United States, the spread of SARS-CoV-2 has also affected residential facilities and rural clinics around the globe. These communities face additional challenges with limited resources and larger logistical obstacles to obtain FFRs.

At the time of publication, shipping used FFRs to localized centers for hydrogen peroxide vapor (HPV, also written as H_2_O_2_ vapor) decontamination was only available in very limited locations in the United States.^1^^–^^3^ HPV decontamination is a U.S. Food & Drug Administration-approved method for N95 mask decontamination, and manufacturing and deployment of these systems is currently underway. However, the operational and coordination challenges associated with even localized deployment of HPV centers for N95 decontamination are significant. This is evidenced by contemporary reports of HCWs being issued FFRs for continuous (re)use over week-long time periods.^4^^–^^6^ Even many months after this pandemic entered the global arena, many locations around the world and in the United States are still struggling to provide adequate PPE for HCWs and other staff at high risk.^7^^,^^8^ Additionally, Nebraska Medicine has initiated a U.S. Centers for Disease Control and Prevention (CDC) approved on-site ultraviolet germicidal irradiation (UVGI) decontamination system for FFR mask decontamination.^9^ UVGI has been demonstrated to be effective at quickly decontaminating FFRs for viruses like the novel SARS-CoV-2 and for multiple cycles of decontamination.^10^^–^^12^ Whereas UVGI decontamination has important limitations as discussed herein, these methods currently are being deployed as emergency procedures during the SARS-CoV-2 pandemic. The Nebraska Medicine protocol uses an operating room UVGI decontamination system for FFR mask decontamination, a system that many smaller clinics, rural hospitals, and residential health care facilities may not have.

We document procedures to build a similar type of UVGI platform using off-the-shelf components from a hardware store and UV-C bulbs that can be obtained through online marketplaces or from biosafety cabinets (class I, II, or III) that are ubiquitously found throughout academic research and industrial centers around the world. This system is scalable; can be created for less than US$50, on site and at the point of need; and leverages resources that are currently untapped and sitting unused in public and private research facilities that have shut down during the COVID-19 pandemic. Health care facilities can obtain this potentially lifesaving UVGI resource with minimal funds by collaborating with research facilities to obtain the UV-C meters and limited availability UV-C bulbs required for UVGI treatment.

We built a UVGI system to decontaminate FFRs for less than US$50 that is easily made on site, is scalable, and uses resources that are unused.

## FFR DEMAND AND NEED DURING THE COVID-19 PANDEMIC

The COVID-19 pandemic is expected to continue to increase the burden on health care providers. As the number of cases increase, health care facilities will continue to be stretched to their limits in terms of supplies and labor. There are 6,146 hospitals in the United States, 5,198 of which are classified as community hospitals; 209 are federal government hospitals, 616 are nonfederal psychiatric hospitals, and 123 other hospitals.^13^ Community hospitals are those that can be accessed by the general public, including short-term general and specialty hospitals, and are classified as rural or urban. There are 3,377 urban community hospitals that serve approximately 106,000 square miles (about 84% of the population), and there are 1,821 rural community hospitals that serve approximately 3.4 million square miles (about 16% of the U.S. population).^14^ These numbers do not include urgent care centers, doctor’s offices, and other non-hospital medical sites involved in the pandemic response.

In contrast, many low- and middle-income countries (LMICs) rely heavily on a limited number of fully resourced hospitals in urban centers with varying degrees of professional health care access in rural areas. Current data on health facilities is difficult to find in many LMICs; however, we can find examples of the resource constraints. For example, Kenya has 842 public and private hospitals serving more than 53 million people.^15^^,^^16^ Only 24 of these facilities are classified as county or national referral hospitals and large teaching or private hospitals. The remainder of Kenya’s more than 11,000 health facilities include smaller clinics, dispensaries, health centers, maternity wards, and nursing homes, not to mention thousands of volunteer HCWs in rural communities. Due to limited supplies of PPE at these facilities, the Government of Kenya recently pleaded with the public to reserve N95 masks for the nation’s HCWs because the entire population was required to wear face coverings in public.^17^

There are currently dramatic shortfalls in protective equipment in countries with relatively robust health care services, like the United States, and these resources are even more precious in resource-limited communities where fewer doctors and nurses are serving larger populations. According to the World Health Organization^18^:

*Africa suffers more than 22% of the global burden of disease but has access to only 3% of HCWs, and less than 1% of the world’s financial resources.*

Losing a single doctor during this pandemic can have a detrimental impact on already strained health care systems across the continent.^19^ Even in the United States, rural clinics and hospitals serve patient populations sometimes across hundreds of miles, and in some areas, there is a single doctor for several thousand square miles.^20^ Ensur-ing HCWs around the world are protected as best as possible is not only ethical, but imperative.

Challenges exist for both urban and rural health care facilities globally. Although urban hospitals often have access to more resources due to the larger population they serve, they experience a strain on their resources during a pandemic precisely because of the significantly larger numbers of people they need to urgently treat. Conversely, rural clinics and health care facilities around the globe often have less funds to operate and face additional logistical challenges to provide patients access to care. Facilities cannot afford to have staff become ill and lead to a decrease in the number of HCWs to treat patients, and therefore need proper FFRs to protect themselves. Additionally, HCWs can potentially spread infection if they are not properly equipped with essential FFRs or if they are forced to reuse potentially contaminated masks. Many HCWs are currently facing the options of not wearing an essential mask, constructing makeshift FFRs with limited efficacy, or reusing a soiled mask—the majority are choosing the latter.^8^^,^^21^^,^^22^ There is an unprecedented worldwide shortage of lifesaving equipment that our HCWs need to continue serving their communities safely. In addition, essential personnel working in pharmaceuticals, dentistry, custodial services, delivery services, and law enforcement also require protection while they keep operations afloat. A global shortage of FFRs is expected to persevere due to supply chain challenges, especially for 1 essential component: the melt-blown polypropylene fabric material that filters infectious diseases like the SARS-CoV-2 during inhalation by the wearer.^23^ As the virus continues to spread into both overburdened and underserved health care systems around the globe, already limited FFR supplies are in extremely short supply. Distributed systems for N95 decontamination are needed to keep up with demand.

Because of PPE shortages, many HCWs are facing the option of not wearing a mask, constructing a makeshift FFR, or reusing a soiled mask.

The CDC estimated that a 42-day influenza outbreak in the United States, which represents just 4.25% of the global population, could require more than 90 million FFRs for HCWs alone.^24^ This would scale to almost 800 million FFRs in a year. A model of a hypothetical influenza pandemic predicted 1.7 to 7.3 billion respirators would be required if only 20%–30% of the U.S. population were to be infected.^25^ This does not account for non-HCWs, such as law enforcement officers and other essential personnel, who may require respiratory protection. Given the uncertain nature of this pandemic and demonstrated logistical challenges in obtaining adequate resources, it is reasonable to assume that need will far exceed the value given in this projection and that demand will only grow.

## FFR DECONTAMINATION AND REUSE

FFRs are designed and manufactured for single-use applications. Depending on the specific country, FFRs are named differently as masks with the following distinction as N95 (United States), FFP2 (Europe), KN95 (China), P2 (Australia/NZ), Korea 1^st^ class (Korea), or DS (Japan). There is slight variability to the mask filtering specification depending on government regulations, but these masks are all expected to function similarly.^26^ Each mask is composed of polypropylene fibers to create a physical barrier based on pore size and leverages the electrostatic charge of the material to improve filtering of aerosolized particles such as SARS-CoV-2 viral particles. The inability to scale FFR manufacturing at the rate needed to meet current demand during the COVID-19 pandemic has necessitated the reuse of N95 respirators among HCWs. Work has shown that pathogens such as viruses can contaminate and exist for extended periods of time on the outer surface of FFRs.^27^ Beyond the risk to HCWs in storing and reusing what is intended to be single-use PPE, other at-risk patients could be exposed to the virus when consulting with a HCW who is reusing their PPE that was previously used with a patient who is positive for COVID-19. It should be noted that repeated redonning FFRs alone poses serious risk to the user due to loss of strap elasticity, nose fit, and therefore, mask integrity. Due to need, redonning is already occurring, and these masks have the potential to be contaminated with viral particles, risking further spread of the virus.

The CDC has suggested a method of FFR reuse by issuing each HCW 5 FFRs. Upon completion of a shift, the FFR is placed in a paper bag and redonned after sitting in that bag for 5 days.^28^ This time period was determined based on data that suggests that SARS-CoV-2 can exist on surfaces for as much as or longer than 72 hours. This allows a HCW to cycle through their own previously used masks; however, there are still a variety of problems with this method. Potentially contaminated masks must be stored for long periods of time, and often HCWs do not have the proper space to do this. Masks may also be heavily soiled or moist from the prolonged wear, which would preserve the virus for longer durations, and it has not been rigorously tested whether 5 days is enough time to decontaminate masks under these conditions. HCWs and other essential personnel are desperate for another solution to decontaminate masks effectively, cheaply, and more quickly.

Decontamination of FFRs must be considered carefully because improper decontamination can also give users a false sense of security in addition to compromising mask integrity. A variety of options have recently been developed to allow for decontamination between uses including HPV decontamination, UVGI treatment, and the applications of heat/humidity/washing.^29^ The recently established N95DECON website (https://www.n95decon.org) gives a summary of these methods, including current understandings and limitations to consider for each method. The U.S. Food & Drug Administration approved hydrogen peroxide-based decontamination offered by a U.S. company, Battelle. Although it is a highly effective resource, the procedure relies on shipment of contaminated masks to Ohio, where the company is based, for decontamination^30^^,^^31^ and/or the production of such HPV decontamination equipment for regional deployment. The ability for health care facilities and health workers to decontaminate their own masks in minutes, as opposed to days, is a great advantage of using UVGI.

Other methods of decontaminating FFRs, although effective, are time-consuming and costly, particularly for facilities with limited funding and resources.

Importantly, UVGI is listed by the CDC as an appropriate method of FFR decontamination and provides key considerations when using this method of decontamination.^28^ Successful implementation of UVGI in a hospital setting is already being used by Nebraska Medicine.^9^ Unfortunately, their system requires 2 surgical suite UVGI towers, with each costing in excess of US$20,000, which not all health care facilities have available.

Herein, we developed a UVGI lamp setup that provides the capability for health care facilities and local regional centers that do not have access to operating room UVGI towers to implement their own N95 mask decontamination system. These health care facilities can use our proposed low-tech UVGI lamp, along with the work flow developed by Nebraska Medicine, to decontaminate FFRs in their own centers. Our UVGI lamp is accessible, inexpensive, requires little expertise to construct and operate, and repurposes existing UV-C bulbs not currently in use. The system takes advantage of common parts available at any hardware store. Once implemented, this method allows for high throughput and quick decontamination cycles that should allow for safer redonning of FFRs.

## FFR DECONTAMINATION USING UVGI

UVGI systems have been used throughout the health care industry to decontaminate work environments such as surgical suites, equipment, and ambulances. Single-stranded RNA (ssRNA) viruses, like SARS-CoV-2, are especially susceptible to UV decontamination.^10^ Previous work has shown that UVGI systems can also be used to decontaminate FFRs by reducing the viability of the influenza virus, also an ssRNA virus, by 3 log.^11^ A very recent small study has shown that UV is capable of decontaminating N95 mask fabric contaminated with SARS-CoV-2.^32^ There is variable effectiveness of UVGI depending on the mask manufacturer, the different materials of the mask (polypropylene filter versus rubber strap), and the medium in which the virus resides (in liquid, in air, on surface). UVGI decontamination also runs the risk of damaging the FFR materials, which can compromise the integrity of the mask and its usefulness in filtering particles and acting as an effective piece of PPE. However, a variety of studies have looked at the effect of UVGI on mask integrity,^33^^,^^34^ even with repeated exposure,^12^^,^^29^^,^^35^^–^^37^ and have found no significant increase in viral penetration, nor decrease in mask stability, even at UVGI doses >10,000 times the required dose to effectively reduce influenza infectivity.^38^ In other studies, 3 UVGI cycles of 1.6–2.0 mW/cm^2^ for 15 minutes did not cause significant changes in respirator fit,^35^ and there was no change in filtration performance. However, using UVGI to decontaminate FFRs requires careful monitoring of UV dosage and the number of times a single mask is decontaminated to minimize damaging the mask’s integrity. The current literature related to UVGI decontamination of N95 masks, mainly in the context of influenza, is summarized (Table). Importantly, the CDC has approved the protocol from Nebraska Medicine for UVGI decontamination during the COVID-19 pandemic.

**TABLE.** Summary of Literature Using Ultraviolet Germicidal Irradiation for Filtering Face Respirators Decontamination

**First Author**	**Year**	**Dose**	**Time**	**Dose**	**Repeated**	**Total Accumulated Dose**	**Distance**	Bulb Specs	**Effectiveness**	**Room Conditions**	**Fit/Degradation**
Tseng^10^	2007		5–255 sec	2.51–6.50 mJ/cm^2^	1×	2.51–6.50 mJ/cm^2^	30.5cm	4 × 8 W	Kills 99%, decreased effectiveness at higher humidity	21–28°C, 55% & 85% RH	
Viscusi^29^	2009	0.18–0.20 mW/cm^2^	15 min/side	324–360 mJ/cm^2^	3×	0.97–1.08 J/cm^2^		40W		21°C, 50% RH	No observable physical changes, filtration performance not affected, no noticeable changes in airflow resistance
Bergman^36^	2010	1.8 mW/cm^2^	15 min	1.62 J/cm^2^	3×	4.86 J/cm^2^	25 cm	40 W		21°C, 50% RH	No observable physical changes, no significant change in penetration
Heimbuch^33^	2011	1.6–2.2 mW/cm^2^	15 min	1.44–1.98 J/cm^2^	1×	1.44–1.98 J/cm^2^	25 cm	80 W	>4 log reduction		Effective decontamination against droplet and aerosolized influenza challenge, no change to fit after decontamination
Bergman^35^	2011	1.6–2.0 mW/cm^2^	15 min	1.44–1.98 J/cm^2^	3×	4.32–5.94 J/cm^2^		40 W	>4 log reduction	21°C, 50% RH	Fit not significantly affected after 4 fit tests, no degradation
Viscusi^34^	2011	1.6–2.0 mW/cm^2^	15 min/side	2.88–3.96 J/cm^2^	1×	2.88–3.96 J/cm^2^		40 W		21°C, 50% RH	No changes in fit, odor detection, comfort, or donning difficulty with UVGI- masks were redonned 5×
Lore^37^	2012	1.6–2.2 mW/cm^2^	15 min	1.44–1.98 J/cm^2^	3×	4.32–5.94 J/cm^2^	25 cm	2 × 15 W	>4 log reduction		Decontamination methods did not significantly degrade filter performance at 300 nm particle size
Lindsley^38^	2015		n/a	120–950 J/cm^2^	1×	120–950 J/cm^2^	6.2 cm	2 × 15 W		27°C, 25% RH	No significance change in flow resistance, decreased penetration strength and strap breaking strength at very high exposure
Mills^11^	2018		60–70 sec	1.1 J/cm^2^	1×	1.1 J/cm^2^	1 m	8 × 0.39 W/cm^2^	>3 mean log reduction, straps less effectively decontaminated	21°C, 50% RH	Masks oversaturated with virus in physiologically relevant solvents sebum and mucin, ultraviolet germicidal irradiation levels fully decontaminated masks, statistically significant reduction in virus viability
Heimbuch^12^	2019	16–18 mW/cm^2^	60–70 sec	1.0–1.2 J/cm^2^	20×	20 J/cm^2^	1 m	8 × 0.39 W/cm^2^	>3 mean log reduction, straps less effectively decontaminated, tested on SARS-CoV-1	22.5°C	No meaningfully significant effect on fit, air flow resistance, or particle penetration for 15 models tested, some models had significant effects due to donning/doffing cycles
Card^42^	2020	0.1 mW/cm^2^	20 min/side	240 mJ/cm^2^			60.6 cm	30 W			
Lowe^9^	2020	0.2 mW/cm^2^	5–6 min	300 mJ/cm^2^			10 ft	16x			

Abbreviations: J, Joules; mJ, milliJoules; mW, milliwatts; RH, relative humidity; W, watts.

UVGI inactivates viruses by damaging their nucleic acids and, to a lesser extent, their protein capsid. UV-C wavelengths (100–280 nm) have the highest decontamination efficiency because the maximum absorption wavelength is 260 nm and 280 nm for nucleic acids and proteins, respectively.^39^ Although little is known about the novel SARS-CoV-2, comparisons between SARS-CoV-2 and SARS-CoV-1 can be helpful in estimating conditions in which SARS-CoV-2 may persist. As mentioned, ssRNA viruses, like SARS-CoV-2, are the most susceptible type of virus to UVGI, which is important for understanding the potential success of UVGI treatment.^10^ SARS-CoV-2 can persist on plastic and stainless steel for up to 3 days, although at 72 hours virus titer had decreased 3-fold, and no viable SARS-CoV-2 was measured on cardboard after 24 hours.^40^ How long this virus can remain viable on PPE has yet to be studied and is likely a function of room humidity, contaminating fluids, mask materials, and construction. Because FFRs from different countries of origin use the same polypropylene material to create the mask filter, it is expected that UVGI would perform similarly on the various FFRs. As the following section details, the correct wavelength range is important for the treatment system and must be validated using a UV-C meter to ensure effective dosing in each setup.

## PROPOSED SOLUTION: INEXPENSIVE, SCALABLE, AND ACCESSIBLE UVGI SYSTEM FOR FFR DECONTAMINATION

We propose a collaboration between public and private research institutions and health care facilities to increase the access to UVGI decontamination. Currently, UV-C bulbs required for UVGI are in limited supply; however, most laboratory biosafety cabinets (BSCs) are equipped with UV-C bulbs, and thousands of these bulbs are currently sitting idle, as research has been mainly halted to slow the spread of the virus. Additionally, many research institutions recommend other chemical means of decontamination, thus additionally leaving UV-C bulbs idle. These bulbs also may be available in existing health care facility settings (e.g., tuberculosis wards) where they can be used for decontamination via passive fixtures or air handling units.^41^ Although others have proposed decontaminating FFRs within these BSCs directly,^42^ this requires the masks to be transported to and from the research institutions and staff to run the decontamination cycles. Our approach advances previous work and that from Nebraska Medicine and allows medical sites to create their own decontamination system, using the idle bulbs, by retrofitting or creating custom light fixtures with off-the-shelf parts available at any retail hardware store.

### Building a Mask Decontamination System

We have developed step-by-step instructions to create UVGI light fixtures that can be downloaded from our website: https://www.gleghornlab.com/uvgi-decontamination. There are multiple options for fixture assembly, depending on availability of materials, that include:

1. Modifying a premade commercial light fixture to fit new or existing UV-C bulbs:
  - Using an existing ceiling light fixture
  - Modifying a hanging ceiling fixture
2. Creating a custom fixture from off-the-shelf parts to fit new or existing UV-C bulbs

Because the number and type of bulbs available will vary, we created an easy to follow protocol to create this lamp using almost any common BSC UV-C bulb. The design plans we developed are not limited to BSC UV-C bulbs, and we provide details to adapt them to any UV-C bulbs. The digits printed on a UV-C bulb provide the information necessary to the end user to adapt these bulbs to create these custom UVGI lamps. For example, a bulb labeled “G30T8”: G stands for germicidal, 30 is the wattage, and T8 represents the size of the bulbs and pin geometry. For bi-pin bulbs, T5 has a 5/8 inch diameter, T8 has a 1-inch diameter, and T12 has a 1.5-inch diameter. This information is important to match to the correct fixture/bulb holder.

### Determining UV-C Exposure Time Required for UVGI Decontamination of FFRs

**FIGURE 1.**
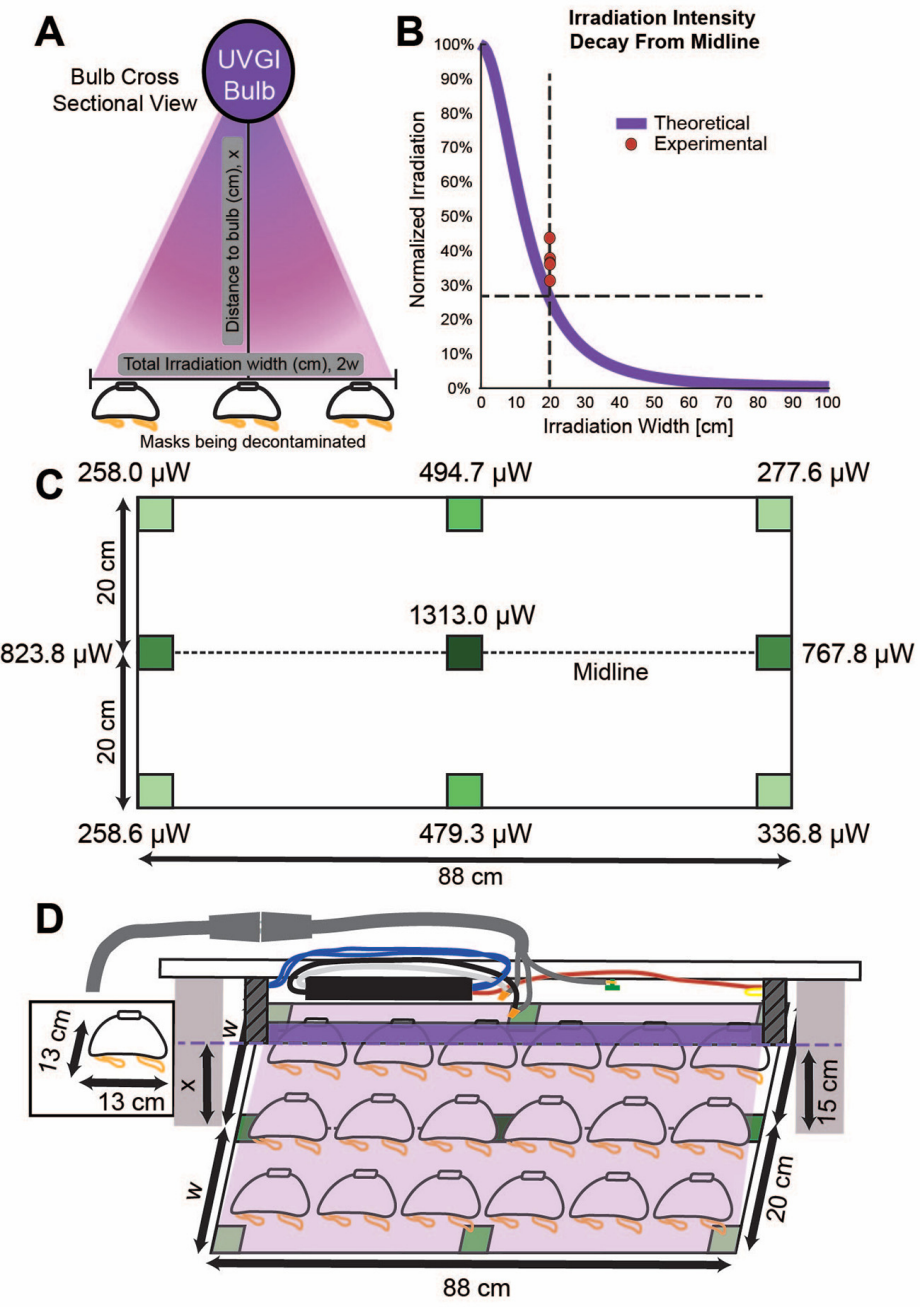
(**A**) Schematic demonstrating the geometric variables that determine ultraviolet germicidal irradiation area. (**B**) Experimental measurements of ultraviolet intensities are well described by a theoretical model to calculate ultraviolet irradiation. (**C**) Ultraviolet-C intensities were measured in an irradiation area 88 cm x 40 cm and the midline was 15 cm from the bulb source. As expected, ultraviolet intensities decay with increasing distance from the bulb, with the lowest intensities measured at the corners of the array. (**D**) Cartoon of an example table top ultraviolet germicidal irradiation setup. Abbreviation: UVGI, ultraviolet germicidal irradiation.

Similar studies analyzing the dosages required for proper UVGI decontamination and reuse of FFRs report an optimal UV-C dose of approximately 300 mJ/cm^2^.^9^ To determine UV dosage, design geometries, scalability, and N95 mask decontamination throughput of our designs, we compared a model of irradiation^43^ (measure of UV-C intensity per area) and compared it to measured values of UV-C irradiation from our modified fixture. UV-C intensity on a fixed plane from cylindrical source is nonlinear and is a function of bulb characteristics (length, wattage, radius) and distance to the bulb (Figure 1A). For a single bulb, the highest irradiation will be achieved along the midline and will rapidly decay with increasing irradiation width (Figure 1B). We used a UV meter (attenuation λ=254 nm) to measure the irradiation area at discrete points using our modified fixture outfitted with a single UV-C bulb (G30T8 with 13.4 W UV output) taken from an existing BSC. The bulb was 88 cm long and placed 15 cm above the UV-C meter sensor. We tested areas along the midline and 20 cm away from the bulb’s midline in either direction, with the sensor flat on the table surface at all locations tested. The highest irradiation was along the midline directly under the bulb and the UV intensities decay, as expected, with increasing irradiation width (Figure 1B, C). Importantly, these measurements demonstrate 2 points. Firstly, the model is a conservative prediction of the intensity distribution, and thus can be used to inform bulb configuration geometries described herein. Second-ly, that the bulb intensity is not uniform along its length. Both of these details reinforce the need for user-developed designs and configurations to be validated with UV-C meter measurements to determine the minimum values of UV intensity over the exposure area to calculate exposure times needed in individual configurations.

To determine UV dosage, optimal design geometry, scalability, and mask decontamination throughput, we measured values of UV-C irradiation from our modified fixture.

We measured a new, unused N95 mask (model 7048, 3M) to be 13 cm wide, and therefore we could decontaminate an array of 6 x 3 masks (Figure 1D). We recommend using a reflective surface such as a stainless steel table or table coated with simple aluminum foil when irradiating on a table surface to maximize irradiation intensities due to reflection.

To calculate the UV-C exposure time, t, in seconds, s, needed for treatment, as:

$$t=\frac{D}{I}\times 1000$$

where *D* is the desired dosage value and *I* is the lowest measured irradiance value from the UV-C meter within the irradiation area.

For an array of 6 x 3 masks, using a single bulb, we use the lowest measured UV-C value over the exposure area (258 µW/cm^2^, from Figure 1C) to determine the irradiation time. For our measured setup, the desired dosage (D) is 300 mJ/cm^2^ for SARS-CoV-2^9^, irradiance value (I) is 258 µW/cm^2^, and exposure treatment time (t) is 1162.8 seconds or 19.4 minutes for each side of the mask.

$$t=\frac{300}{258}\times 1000=1162.8$$

Because the different UV-C bulbs able to be used in this proposed system will have parameters depending on the specifications such as size and age of the bulb, the end user of their own specific UVGI system will need to validate the UV-C values. This should be done using a UV-C meter to determine actual irradiation areas and irradiation times to properly decontaminate the FFRs.

### Optimizing UVGI Decontamination

#### Minimize Irradiation Deficits on Mask Surface

**FIGURE 2.**
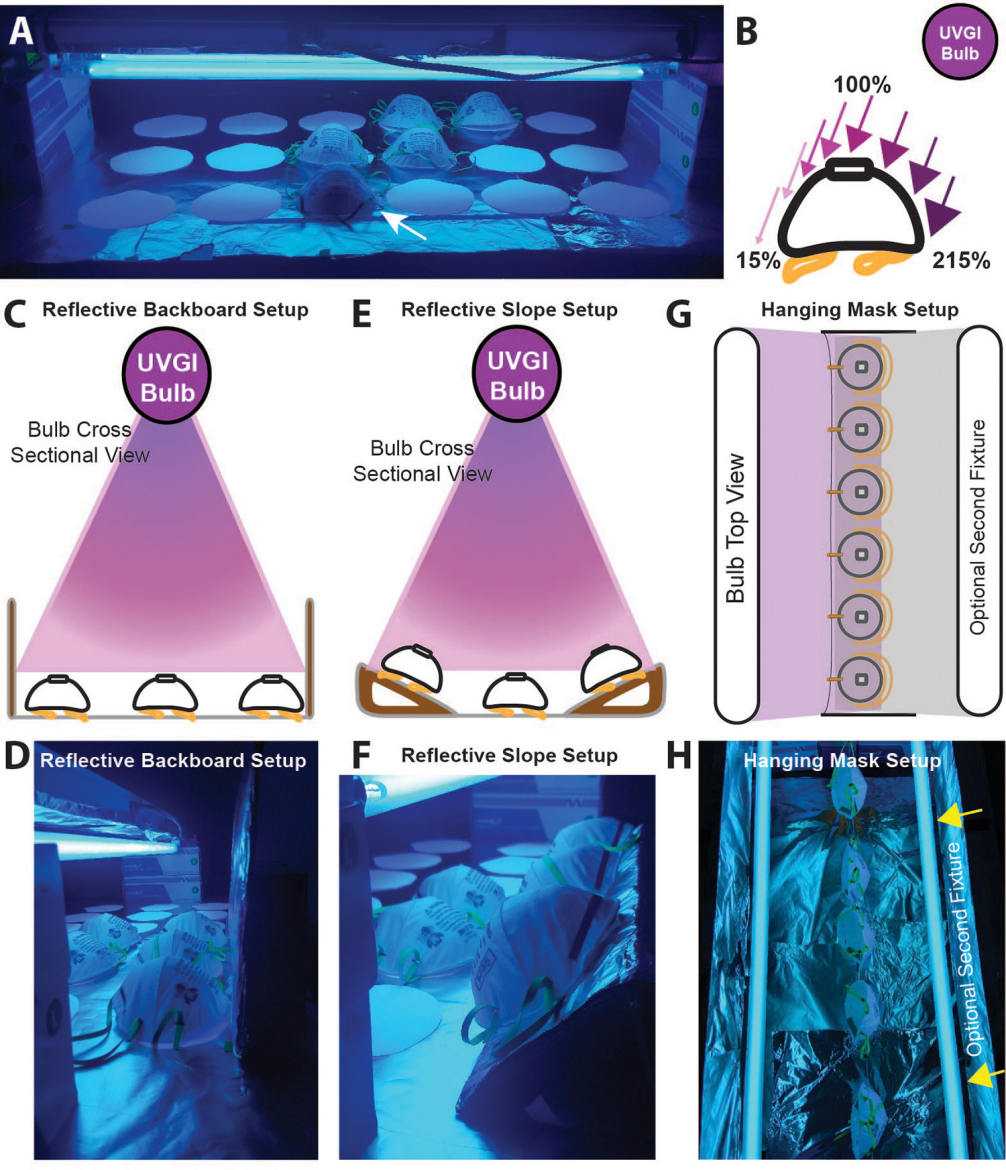
Multiple configurations can improve irradiation on the mask surface. (**A**) Image showing the table top setup of our custom ultraviolet germicidal irradiation. White circles on the table top demonstrate the foot print for placement of masks to be decontaminated within an 88 cm x 40 cm irradiation area. A shadow is visible on the far surface of the mask (white arrow) indicating decreased exposure due to the curvature of the mask and angle of the ultraviolet rays. (**B**) The far surface of the mask on the outer rows of our irradiation area, the mask region furthest from the bulb, receives 15% of the ultraviolet intensity compared to the top of the mask. (**C, D**) By adding a reflective backboard of aluminum foil wrapped cardboard to the edge of the irradiation area, the ultraviolet rays can be reflected to increase the irradiation exposure to the far surface of the mask. (**E, F**) Alternatively, by creating a sloped surface for the masks at the edge of the array, mask surfaces can be more aligned toward the light source resulting in more uniform irradiation intensities across the surface. (**G, H**) Another decontamination setup includes suspending or hanging the masks. If there is access to a second, optional fixture, this allows for irradiation of both sides of the mask simultaneously.

It is important to note that masks placed too close together can create shadows that prevent effective UV decontamination. Additionally, when irradiating masks on a table surface without reflective surfaces, the dome shape of the masks inherently makes it difficult to decontaminate the surfaces on the far side of the mask relative to the position of the light source (Figure 2A, B). To combat this issue, we devised 2 solutions: (1) place a reflective “backboard” at the edge of the array, and (2) place a reflective wedge underneath the mask on the edge of the array to angle the masks toward the light source. A simple wall made of aluminum foil covered cardboard was placed at the edge of the irradiation area (Figure 2C, D). The UV light was able to be reflected off this surface to achieve greater irradiation on the far surfaces of the mask. Using this method, UV-C intensity measured on the mask surface was 63% of the intensity measured on the front mask surface. In the second approach, a reflective wedge was created by wrapping aluminum foil around a folded piece of cardboard (Figure 2E, F). This sloped surface decreases the effect of the domed shape of the mask by orienting the mask in the direction of the light source to decrease distance from the bulb and prevent shadows caused by the mask surface relative to the bulb position. Using this method, edge region intensities were 79% that of the measured intensities on the front surface of the mask. However, this modification, in general, places the mask closer to the light source, increasing the overall irradiation intensity by 298%. Such solutions are simple and important to reduce shadows from occurring. Calculated exposure times should be based on the lowest irradiation value at the edge of the irradiation area as measured on the edge of the mask.

#### Reduce Distance to Fixture

If we reduce the distance to the bulb, the measured irradiation on the masks will increase non-linearly and will reduce the time needed to achieve a dose of 300 mJ/cm^2^ per side of the mask. Further increases in mask decontamination throughput could be achieved by adding a second light fixture which will allow exposure to both sides of the mask simultaneously, similar to the Nebraska Medicine configuration, reducing the total time for irradiation in half (Figure 2G, H).

#### Add Additional Fixtures

**FIGURE 3.**
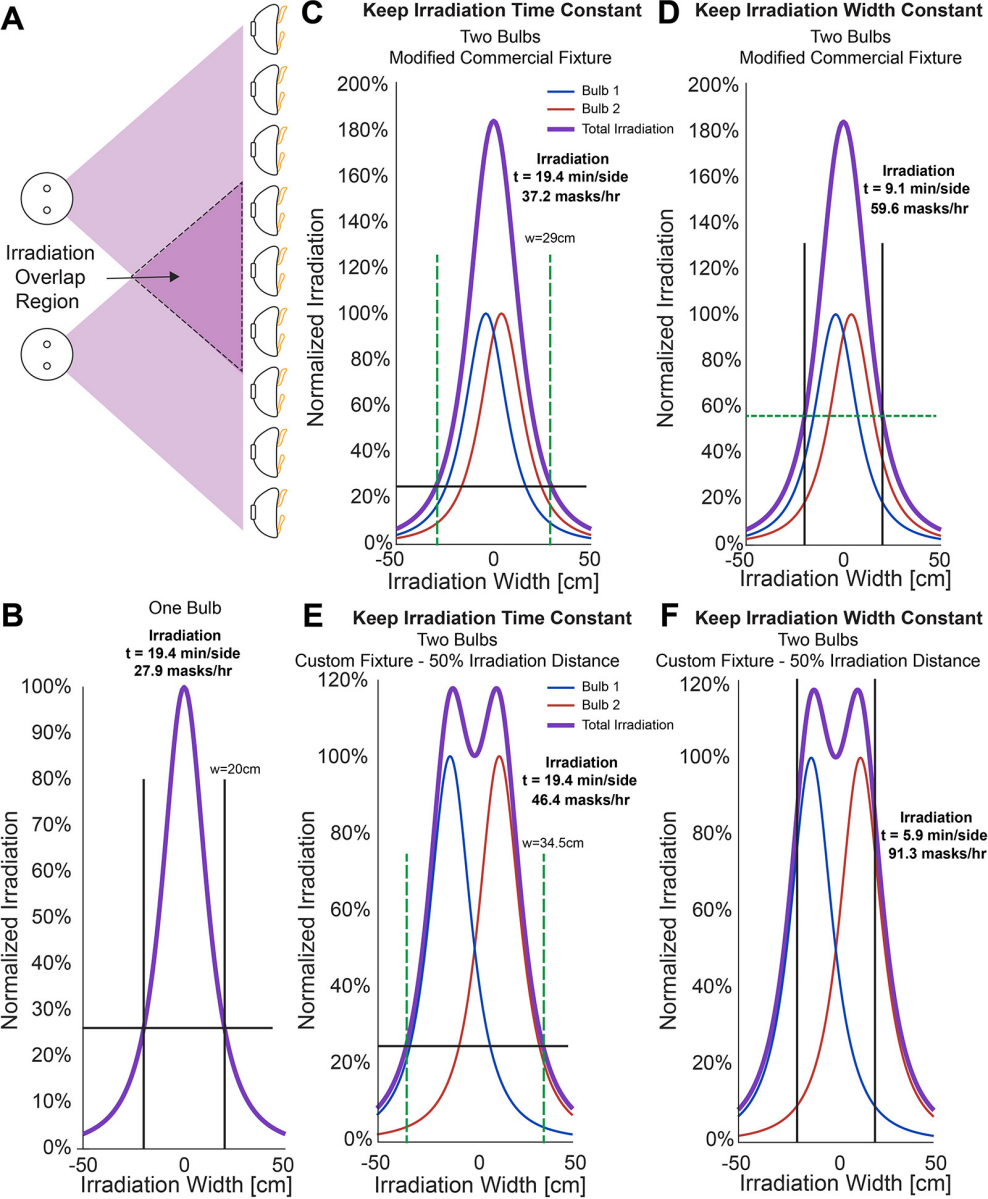
Irradiation time can be optimized by placing 2 fixtures in close proximity. (**A**) The ultraviolet light from both fixtures will overlap to create an irradiation overlap region where the ultraviolet intensities are additive. (**B**) One bulb will create a single maximum irradiation peak; varying the distance between 2 bulbs creates different irradiation and decontamination scenarios which can be modeled to find the optimal conditions. (**C, D**) Two bulbs modeled in very close proximity; or (**E, F**) modeled at an optimal distance can increase the irradiation area if the irradiation time is fixed or decrease the irradiation time by increasing the overall exposure within the original irradiation area.

Additional light fixtures can also be placed in parallel to increase the total irradiation area. If placed in close enough proximity, the 2 light fixtures will create an irradiation overlap region which will increase the irradiation intensity to reduce the time needed to decontaminate (Figure 3A). By modeling the theoretical irradiation curves following thermal radiation view factors,^43^ we can determine the effects of adjusting the bulb spacing on the mask decontamination throughput capacity. The 100% irradiation value used in our model was delegated as the highest measured UV-C intensity that occurred in the center of the array (Figure 1C). For our single bulb setup, using an irradiation width of 20 cm, processing 18 masks in an array, the decontamination time is 19.4 minutes per side, and thus can decontaminate 27.9 masks/hour (Figure 3B). This time is determined by the lowest intensity measured in our array where the intensity is approximately 26% that at the midline. However, if we use a modified commercial light fixture outfitted with 2 UV bulbs, the bulbs are closely spaced resulting in high overlap in UV light and the peak intensity of UV is notably increased (Figure 3C, D). This geometry can either produce a larger irradiation width for the same exposure time from 20 cm to 29 cm to increase mask throughput (Figure 3C), or we can keep the irradiation width the same and yield a higher UV intensity (Figure 3D). A higher UV intensity decreases the exposure time req-uired and increases mask throughput. Although counterintuitive, Figure 3D demonstrates highest mask throughput (59.6 masks/hour compared to 37.2 masks/hour) by keeping the irradiation field constant (i.e., fewer masks at once) and having a higher UV intensity to decrease exposure time. These measurements and calculations are for a single modified, existing, commercial 2-bulb light fixture.

Although having 2 bulbs in a commercial fixture increases throughput compared to 1 bulb, taking advantage of off-the-shelf components to build a custom fixture has significant advantages. Specifically, users can optimize bulb spacing to increase the uniformity of the irradiation field. If 2 bulbs are spaced at the 50% irradiation width intensity for 1 bulb, the UV intensities are additive, which creates a more constant irradiation field (Figure 3E, F). Using this configuration, the irradiation width is considerably increased from 20 cm to 34.5 cm for a mask exposure time of 19.4 minutes/side. This generates a mask decontamination throughput of 46.4 masks/hour (Figure 3E). How-ever, similar to findings from the off-the-shelf fixture, keeping the irradiation area constant to take advantage of the higher UV intensities will increase mask throughput more substantially. This approach reduces decontamination time to 5.9 minutes/side and yields a throughput of 91.3 masks/hour for a system with lights only on 1 side of the masks (Figure 3F). This represents a greater than 3-fold increase in mask processing from 1 UVGI bulb. If a system is created with simultaneous exposure to front and back sides of the masks, using 2 UVGI bulbs on each side, a user could process 182.6 masks/hour. Choosing an optimal setup will all depend on the resources at hand, such as fixtures, bulbs, and treatment environments. The end user will need to validate the UV-C values, using a proper UV-C meter, once the desired configurations of UVGI bulbs are in place to determine actual irradiation areas and irradiation times to properly decontaminate the FFRs. A significant advantage of this system is the potential for parallelization of numerous UVGI light fixtures to simply scale the N95 mask treatment throughput.

### Approximate UVGI Fixture Assembly Costs

**FIGURE 4.**
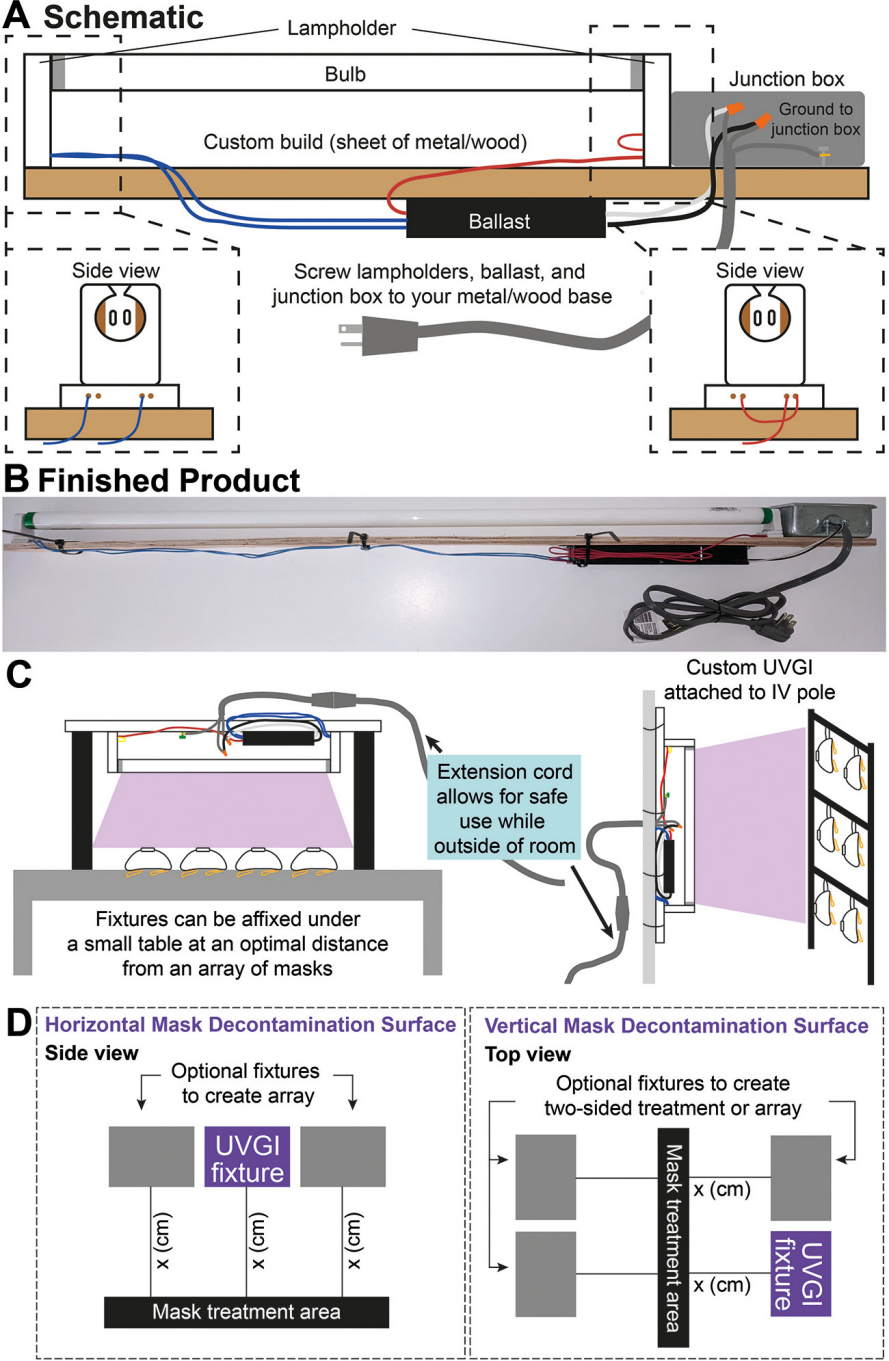
An easy, affordable, custom ultraviolet germicidal irradiation system can be built with off the shelf parts. (**A**) Cartoon representation of our custom built ultraviolet germicidal irradiation fixture. (**B**) Finished product of our custom ultraviolet germicidal irradiation fixture. (**C**) Fixtures can be placed over tables or placed vertically by attaching it to health care equipment such as an intravenous pole. (**D**) Example arrays showing how a user might assemble a horizontal mask decontamination surface or a vertical surface which would allow for irradiation on both sides of the mask pending the availability of additional ultraviolet germicidal irradiation bulbs.

This system leverages affordable components and resources already available and distributed across the country in a variety of different research centers. Using our approach to modify commercial fixtures from the hardware store, 1-bulb, 2-bulb, and 4-bulb UVGI light fixtures can each be constructed for less than US$25, US$30, and US$45, respectively. Each custom built fixture (Figure 4A, B) can be constructed for less than US$21 for a 2-bulb configuration and less than US$36 for a 4-bulb configuration. These costs do not include the cost of the bulb itself which we believe could be obtained by leveraging collaborations with research institutions to use UV-C bulbs that might otherwise be sitting idle. To implement this UVGI light source, a UV-C meter should be used to provide an accurate measurement of irradiance (µW/cm^2^) at the position of the FFRs will be placed away from the UVGI system.^4^^,^^23^ We recommend using a similar workflow and arrangement that Nebraska Medicine developed with their UVGI light towers. Both modified light fixtures or custom fixtures can be easily propped over the decontamination surface by simply resting on boxes (Figure 2A) or affixed to common items in a medical facility such as under a table or an intravenous pole to allow customization and adaptability of the decontamination area (Figure 4C). The end user can also adapt the system to decontaminate masks on a horizontal surface and irradiate only 1 side of the mask at a time (Figure 2A, C-F, Figure 4D) or can suspend the masks to create a vertical decontamination system that will allow for decontamination of both sides of the masks simultaneously (Figure 2G, H, 4D). Using these modified light sources adds significant flexibility in the positioning of UV-C systems to generate multiple larger-scale UVGI bulb arrays or several treatment systems in parallel.

Using our approach, UVGI light fixtures can be made for US$25–US$45, excluding the cost of the bulb.

## CONCLUSION

Our system leverages a collaboration between research institutions and health care facilities, but otherwise has minimal associated cost and expertise. Research institutions can donate UVGI bulbs; UV PPE, including goggles and face shields; and the necessary UV-C meter. Health care facilities can assemble the UVGI systems and implement a decontamination protocol such as that established by Nebraska Medicine as an excellent starting point.^9^ Our website (https://gleghornlab.com/uvgi-decontamination) provides the step-by-step downloadable plans to create the UVGI system, as well as helpful graphics to assist end users to determine their required decontamination times based on the measured UV-C output (λ=254 nm) at a defined distance. When working with our proposed UVGI system, it is also important to use proper UV safety procedures, including avoiding any direct eye or skin exposure to the UV light source.

Whereas these instructions are illustrated using materials available in the United States, this concept has global application. The same bulbs are used in research BSCs, medical settings, and increasingly, water treatment and food safety applications throughout the world. The ability for HCWs to quickly decontaminate PPE on site during any health care emergency could be an important measure to reduce the spread of infectious diseases, especially when time and other resources are limited.

## Notes

**Peer Reviewed**

**First published online:** September 11, 2020
